# High-quality genome resource of a basidiomycetous yeast, *Moesziomyces antarcticus* isolate RS1, isolated from rice seed

**DOI:** 10.1128/mra.00847-23

**Published:** 2024-01-16

**Authors:** William Harris, Gobong Choi, Kiseok Keith Lee, Hyun Kim, Yong-Hwan Lee

**Affiliations:** 1 Department of Agricultural Biotechnology, Seoul National University, Seoul, Republic of Korea; 2 Interdisciplinary Program in Agricultural Genomics, Seoul National University, Seoul, Republic of Korea; 3 Department of Ecology and Evolution, The University of Chicago, Chicago, Illinois, USA; 4 Research Institute of Agriculture and Life Sciences, Seoul National University, Seoul, Republic of Korea; 5 Center for Plant Microbiome Research, Seoul National University, Seoul, Republic of Korea; 6 Plant Immunity Research Center, Seoul National University, Seoul, Republic of Korea; University of Maryland School of Medicine, Baltimore, Maryland, USA

**Keywords:** *Moesziomyces*, *Oryza sativa*, seed fungal community, basidiomycetous yeast, genome

## Abstract

*Moesziomyces antarcticus* (anamorph: *Pseudozyma antarctica*) is a basidiomycetous yeast in the Ustilaginaceae family and is a core member of the rice seed microbiome. *M. antarcticus* RS1 was isolated from surface-sterilized rice seeds. This 18.287 Mb draft genome of *M. antarcticus* RS1 is comprised of a 60.8% GC content and 6,817 protein-coding genes.

## ANNOUNCEMENT

*Moesziomyces antarcticus* (anamorph: *Pseudozyma antarctica*) is a basidiomycetous yeast belonging to Ustilaginaceae. *Moesziomyces* spp. have been detected in the phyllosphere and reported as plant and human pathogens (1–5), plant pathogen antagonists (6–9), saprotrophs decomposing biodegradable polymers and plastics (10, 11), and biosurfactant producers (12). We isolated *M. antarcticus* RS1, a core member of the rice seed microbiome (13), from surface-sterilized seeds of the rice cultivar Nakdong incubated on rice extract agar medium at 23.5°C for 14 days. RS1 was identified based on 99.55% similarity to the internal transcribed spacer sequence of *M. antarcticus* CBS 5955.

For whole-genome sequencing, RS1 was grown on potato dextrose agar medium for 7 days at 25°C and then was inoculated in potato dextrose broth and incubated at 25°C, 200 rpm for 3 days and subsequently collected by centrifugation at 13,000 × *g* for 2 min. Genomic DNA was extracted using the Wizard genomic DNA purification kit (Promega, WI, USA) following the manufacturer’s instructions. DNA was sheared using the Megaruptor 2 (Diagenode, Belgium). DNA fragment size was set to 20 kb, and fragments less than or equal to 10 kb were removed using PippinHT (Sage Science, MA, USA). The sheared DNA was prepared using an SMRTbell library with the SMRTbell Express Template Prep Kit 2.0 (Pacific Biosciences, CA, USA) following the manufacturer’s instructions. DNA sequencing was performed by PacBio Sequel IIe platform (Pacific Biosciences, CA, USA), which was conducted at the National Instrumentation Center for Environmental Management (NICEM), Seoul National University, Korea. The generated sequencing reads achieved peak lengths of 10–25 kbp with an N50 size of 13,726 bp. The PacBio Sequel IIe HiFi sequencing platform generated 11.234 Gbp subreads. Default parameters were used except where otherwise noted. Circular Consensus Sequencing analysis in Single Molecule Real-Time (SMRT) Link (v.9.0.092188) acquired high-fidelity reads (HiFi reads) from the subreads, producing 382,480 HiFi reads (total yield: 4.805 Gbp; mean length: 12.564 kbp) (Table 1). Read QC, error correction, and adapter trimming were conducted using the SMRT Link. RS1’s genome was assembled using NextDenovo (v.2.4.0) (14). The assembled genome consisted of 26 scaffolds with 18.287 Mbp of total length (Table 1). The N50 and N75 sizes of the scaffolds were 738,097 and 541,093 bp, respectively. The gene prediction program AUGUSTUS (v.3.4.0) was used to predict protein-coding genes from the assembled genome with default parameters (15), revealing that the genome contains a total of 6,817 protein-coding genes (Table 1).

**TABLE 1:** Genome assembly statistics of *Moesziomyces antarcticus* RS1

Statistics	*M*. *antarcticus* RS1
Raw reads (bp)	11,234,234,020
HiFi reads^*a*^ (bp)	4,805,603,703
CCS reads^*b*^ (bp)	1,445,099,075
Number of scaffolds	26
N50 (bp)	738,097
N75 (bp)	541,093
Genome size (bp)	18,287,299
GC content (%)	60.8
Protein-coding gene number	6,814

^a^ HiFi reads, high-fidelity reads.

^b^ CCS reads, circular consensus sequencing reads.

*Moesziomyces* spp. produce mannosylerythritol lipids (MELs), which are glycolipid biosurfactants (16). A comparative genome analysis *via* local BLAST search (BLAST+ v. 2.6.0) (17) for the known MEL biosynthesis genes (*Emt1*, *Mac1*, *Mac2*, *Mmf1*, and *Mat1*) on RS1 with *M. antarcticus* ATCC 3488 and other strains confirmed all strains possess MEL-biosynthesis genes. This suggests conserved MEL production in *Moesziomyces* strains inhabiting different ecological habitats. As *Moesziomyces* spp. actively produce MELs under nitrogen-limited conditions (18), RS1 may gain a competitive advantage against microbes during seed maturation. This data will aid further research concerning rice seeds’ core microbes and rice-associated yeasts.

## Data Availability

The genome sequence data and assembled genome of *M. antarcticus* RS1 are deposited in the NCBI Sequence Read Archive under the accession number SRX13462438 and JARWAZ000000000 of the BioProject accession number PRJNA791167.
